# Don’t Go Vaping My Heart: A Case of Vaping-Associated Cardiomyopathy and Lung Injury

**DOI:** 10.7759/cureus.42723

**Published:** 2023-07-31

**Authors:** Emmett Brennan, Alexa Kahn, Morris Kopyt, Abdullah Khan, Ricardo Castillo

**Affiliations:** 1 Internal Medicine, One Brooklyn Health - Brookdale University Hospital Medical Center, Brooklyn, USA; 2 Cardiology, One Brooklyn Health - Brookdale University Hospital Medical Center, Brooklyn, USA

**Keywords:** dyspnea, heart failure with reduced ejection fraction, ejection fraction, e-cigarette associated lung injury, e-cigarette smoking, pneumonitis, vaping, heart failure, cardiomyopathy, evali

## Abstract

The potential adverse effects of electronic cigarette (e-cigarette) use or vaping on pulmonary function have been previously well documented, with the diagnosis of e-cigarette- or vaping-use-associated lung injury (EVALI) has become increasingly common. The potential effects in terms of cardiovascular function and vaping is an area that is less well understood. We present a case of acute respiratory distress and newly onset reduced systolic function in a previously healthy young male.

## Introduction

In recent years, the use of vaping and electronic cigarettes (e-cigarettes) has significantly risen, with a growing number of users each day. Touted as a safer alternative to traditional tobacco smoking, these devices have gained popularity among adults seeking to quit smoking and young individuals. Unlike conventional cigarettes, e-cigarettes are often perceived as harmless due to their enticing flavors and lack of tobacco combustion. However, they also have been shown to cause potential harm to health. Aerosolized compounds emitted from e-cigarettes contain numerous toxic substances, including nicotine, formaldehyde, acrolein, and heavy metals, which can pose significant health threats to users. Research indicates chronic exposure to these chemicals can lead to respiratory problems, cardiovascular disorders, and compromised lung function [1].

E-cigarette companies have come under scrutiny for their marketing tactics that target young audiences, resulting in a concerning rise in vaping among adolescents. E-cigarettes and vaping pens have been found to contain numerous compounds that may cause harm, including glycerol, nicotine, propylene glycol, ethanol, and triacetin [2]. Studies have shown that e-cigarette use acts as a gateway to traditional tobacco smoking, further perpetuating the nicotine addiction cycle [3]. The alarming prevalence of vaping among youth has raised public health concerns, as it threatens to reverse decades of progress in curbing tobacco use and addiction rates [4]. Emerging evidence suggests that vaping may be harmful to the cardiovascular system. The inhalation of toxic compounds found in e-cigarette aerosols can contribute to inflammation and oxidative stress, potentially leading to endothelial dysfunction and an increased risk of cardiovascular disease [5]. Additionally, the use of vaping devices has been linked to severe lung injuries, with cases of e-cigarette- or vaping-use-associated lung injury (EVALI) reported in the United States [6]. While the use of e-cigarette use on pulmonary function has been well documented, its contribution towards heart failure is less well understood. We present the case of a young, previously healthy adult male who developed EVALI and newly onset heart failure with reduced left ventricular ejection fraction after an increase in vaping pen use.

## Case presentation

A 35-year-old male bodybuilder with a history of ascending and descending aortic pseudoaneurysm, partial aortic transection with subsequent thoracic endovascular aortic repair (TEVAR), and lung and liver laceration after being struck by a motor vehicle, presented with lower back pain radiating down his thighs. He also described an inability to stand for prolonged periods that was acute in onset over the previous three days and shortness of breath with intermittent chest tightness.

**Figure 1:**
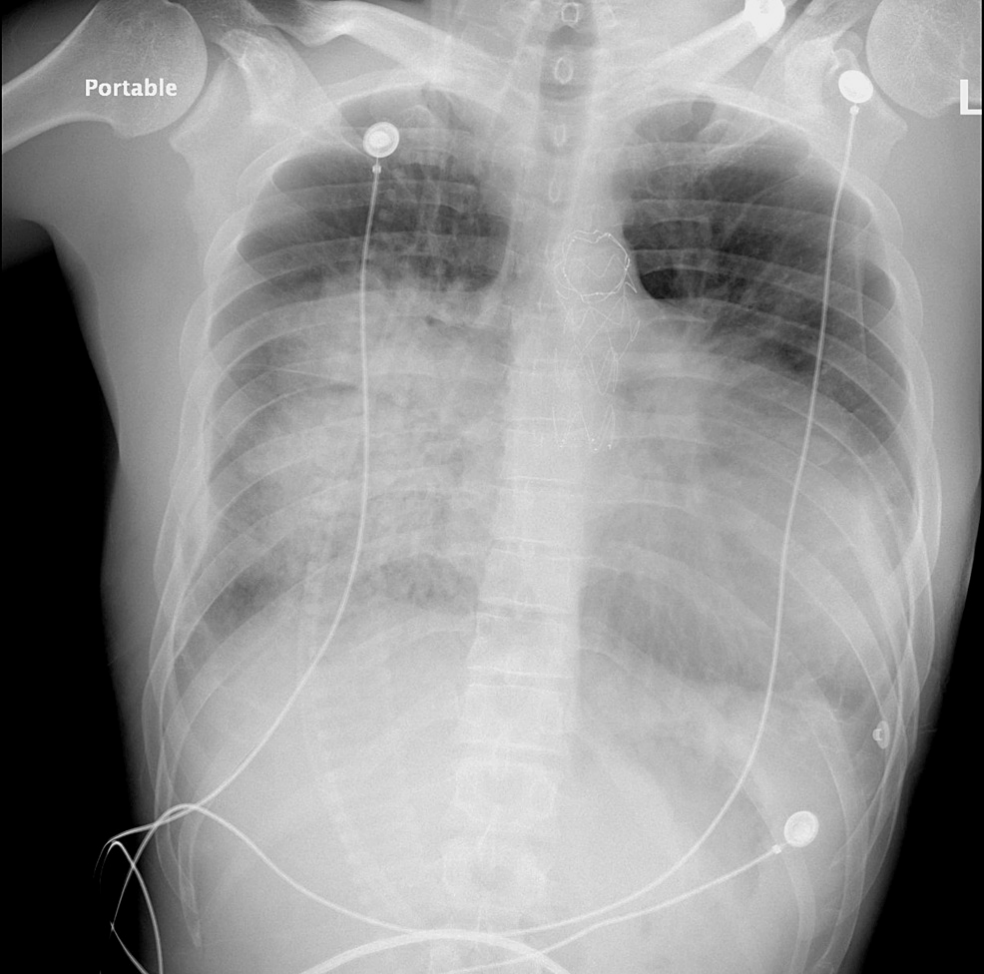
Chest X-ray AP view showing bilateral infiltrates and a left pleural effusion. AP, anteroposterior

The onset of symptoms occurred after engaging in strenuous activity, specifically pushing a motor vehicle for three blocks. The patient endorsed taking multiple workout supplements, including creatine, testosterone, and methasterone for bodybuilding. He denied any cigarette or polysubstance use but did note increased use of his vaping pen in the days before the presentation. On arrival, the patient was hemodynamically stable but later developed hypoxia with oxygen saturation dropping to 85% on room air, leading to the administration of supplemental oxygen. The chest X-ray revealed bilateral pulmonary edema, with a possible infiltrate more prominent on the right side than the left (Figure 1).

**Figure 2:**
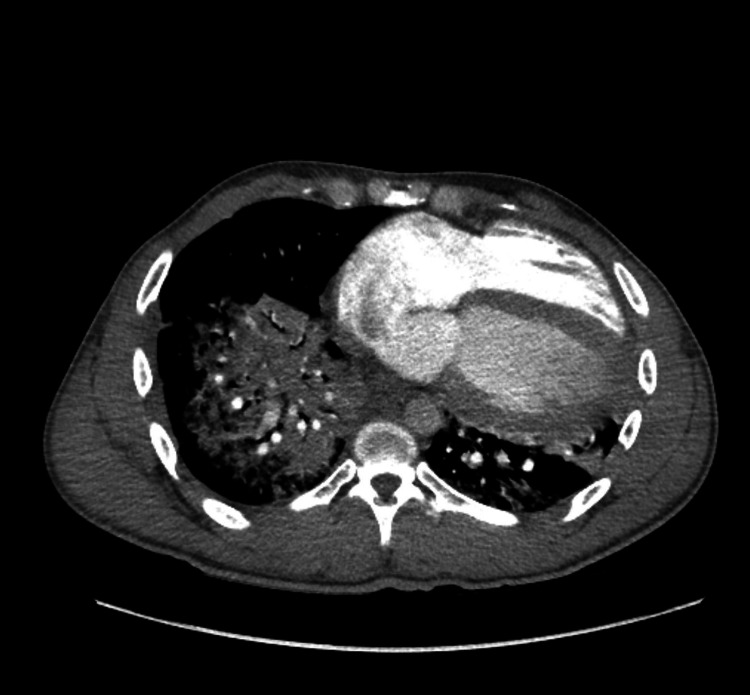
CT angiogram chest axial slice displaying a dense central alveolar pneumonia that extends in bilateral lung fields. CT, computed tomography

The CT angiogram of his chest did not show any evidence of pulmonary embolism; however, it did delineate a very dense central alveolar consolidation involving the right lower lobe and the central right middle and upper lobes (Figure 2).

Lab work was notable for NT-pro BNP of 28,900 pg/mL, Troponin I of 0.271 ng/mL, C reactive protein (CRP) of 14.10 mg/dL, and erythrocyte sedimentation rate (ESR) of 94 mm/hour. The patient was afebrile, without leukocytosis or left shift, and had a negative lactate level, suggesting that a bacterial infection was less likely as the etiology for his presentation. Two sets of blood cultures were negative, and Legionella and Streptococcal pneumonia antigens in the urine were negative. COVID-19 and Influenza swabs were negative, and urine toxicology was also negative.

He was admitted to the telemetry unit and placed on noninvasive positive pressure ventilation (NIPPV) for increased work of breathing. He received corticosteroid steroids for the possibility of bronchiolitis obliterans organizing pneumonia (BOOP). The patient was later noted to have pink frothy sputum, orthopnea, elevated jugular vein distension (JVD), and cough, with complaints of nausea and associated vomiting. He was transferred to the coronary care unit (CCU) for suspected myocarditis or non-ST-segment elevation myocardial infarction (NSTEMI).

**Figure 3:**
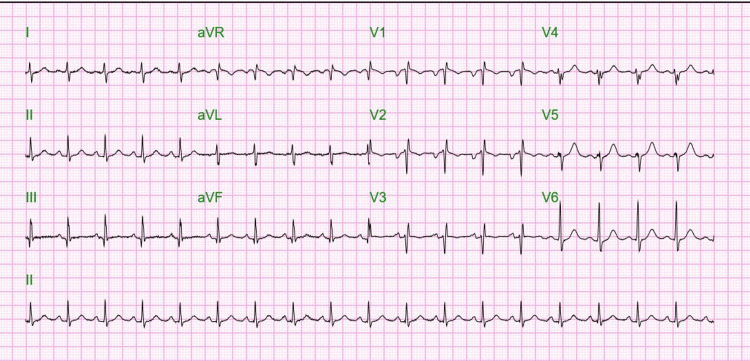
Electrocardiogram showing the S1Q3T3 pattern.

The electrocardiogram revealed an S1Q3T3 pattern (Figure 3), which, in the absence of a pulmonary embolism, indicated right ventricular strain likely due to hypoxemia due to the infiltrate seen on his CT imaging (Figure 3). His echocardiogram was significant for dilated left ventricular with severely reduced systolic function (ejection fraction 32%) and diffuse hypokinesis. A historical echocardiogram before hospital admission demonstrated normal left ventricular ejection fraction. The patient received diuresis with intravenous furosemide and started on guideline-directed medical therapy (GDMT) for new-onset systolic heart failure. A coronary angiogram was done to determine the etiology of systolic heart failure, which revealed normal coronaries and left ventricular end diastolic pressure (LVEDP) of 23 mmHg. 

The patient had an autoimmune workup, which returned as negative. He was seen by the Pulmonology team who advised that his presentation and respiratory imaging could suggest EVALI. His X-ray appearance showed increased aeration of the right lung consolidation seen on admission, and the patient was transferred from BIPAP to nasal cannula oxygen. He showed subsequent improvement with corticosteroids, intravenous (IV) diuresis using furosemide, and goal-directed medical therapy for heart failure. However, he chose to leave the hospital against medical advice before becoming completely independent of supplemental oxygen.

## Discussion

The potential adverse effects of e-cigarette use or vaping on pulmonary function have been previously well-documented, and the diagnosis of EVALI is becoming more commonplace. The potential impacts of vaping on cardiovascular function are an area that is less well understood. We presented a case of acute respiratory distress and new-onset reduced systolic function in a previously healthy young male. There appears to have been an acute temporal relationship between increased vaping pen use and exertion with the onset of symptoms.

Several differential diagnoses were considered in this case. NSTEMI was ruled out as there were no occlusions on the coronary angiogram. Stable ECGs throughout his hospital course make coronary arterial vasospasm unlikely. Pulmonary embolism as a cause of his S3T3Q3 EKG finding was ruled out on CT pulmonary angiogram. This EKG right ventricular strain pattern was likely consistent with a primary pulmonary pathology. The acute course of his symptoms and the absence of low-voltage ECG findings made pathologies such as infiltrative cardiomyopathy unlikely. The CT chest findings appeared to show an infiltrate consistent with EVALI.

In the absence of an alternate etiology for his cardiomyopathy, we consider that his cardiomyopathy may have been precipitated by a lung injury from an e-cigarette or vaping toxicity. This phenomenon has been described in one somewhat similar case [7]. Vitamin E acetate and other volatile hydrocarbons are thought to lead to pneumonitis in cases of EVALI and cause cytokine storms that may impair cardiac function [8]. Similarly sympathetic activation via nicotine in a similar fashion to tobacco smoking can impact cardiovascular function and has been shown to increase the risk of both myocardial infarction in e-cigarette users compared to nonusers and also promote myocardial fibrosis and remodeling [9,10,11]. The role of e-cigarettes on cardiomyopathy and heart failure is an evolving area that requires further study.

## Conclusions

This case report highlights a distressing association between vaping pen use, EVALI, and the development of heart failure in a patient. The presented evidence underscores the need for heightened awareness among healthcare professionals and the general public regarding the potential cardiovascular consequences of vaping.

The emerging popularity of vaping devices has brought about an array of health concerns. In this particular case, the patient's exposure to toxic aerosols through vaping pens appears to have triggered a cascade of events leading to lung injury, which subsequently contributed to the development of heart failure. EVALI, as demonstrated in this case, can have grave implications beyond the respiratory system, with the potential to compromise cardiac function and overall well-being. This emphasizes the interconnectedness of pulmonary and cardiovascular systems and underscores the importance of investigating the potential long-term cardiovascular effects of vaping.

By taking proactive measures and raising awareness, we can hope to curtail the rising tide of EVALI and its potential long-term sequelae. Further studies are needed to evaluate the relationship between EVALI and acute onset decompensated heart failure more comprehensively.
